# Children’s Health: National Children’s Study Begins Recruitment

**DOI:** 10.1289/ehp.117-a19

**Published:** 2009-01

**Authors:** Cynthia Washam

After nearly a decade of planning, the National Children’s Study is finally set to launch. Scientists hope this 20-year nationwide study will unravel the mysteries of some of today’s most significant threats to children’s health.

“The National Children’s Study will help us understand the biological, genetic and environmental factors that make preterm births so difficult to reduce,” says study director Peter Scheidt, medical officer with the Eunice Kennedy Shriver National Institute of Child Health and Human Development (NICHD). “It will help us understand much more about the causes of autism, learning disabilities, asthma, even schizophrenia.”

Impetus for the study began in the 1990s with growing evidence that infants and fetuses are particularly vulnerable to environmental toxicants, and that effects of early exposure may not appear until years later. To assess those risks, the President’s Task Force on Environmental Health Risks and Safety Risks to Children in 1998 proposed a nationwide longitudinal study. Congress authorized the study in 2000, and in fiscal year 2007 appropriated funding of $69 million to begin implementation. Another $110.9 million was appropriated in fiscal year 2008.

The researchers will collect data on the mothers’ pregnancies, including their diets, emotional stress, pregnancy problems, and chemical exposures. The unprecedented study will eventually track the health and development of 100,000 children from birth to age 21. Researchers plan to examine the impacts of the children’s physical, chemical, psychosocial, and biological environments. They also will investigate the role genetics may play in their subjects’ susceptibility or resistance to environmental assaults. Subjects will be enrolled from 105 locations nationwide to provide a population with ethnic, economic, geographic, and racial diversity.

Study enrollment will begin in January 2009 in Queens, New York, and Duplin County, North Carolina, where workers will go door-to-door enlisting women who are pregnant or soon plan to be. In April, recruitment will expand to parts of Pennsylvania, South Dakota, Minnesota, California, Utah, and Wisconsin. By year’s end, researchers hope to have enrolled 1,700 women.

Scheidt and his colleagues view this year’s recruitment as a pilot to refine the study before taking it nationwide in 2010, a recommendation mirrored in a study review by the National Academy of Sciences. “We’ll learn how we can encourage women to stay with us for the entire twenty-one years,” says Scheidt.

The federal agencies coordinating the study include the NICHD, the NIEHS, the Environmental Protection Agency, and the Centers for Disease Control and Prevention (CDC). Universities and hospitals will serve as study centers, enlisting and interviewing subjects, and collecting air, water, dust, and other environmental samples, as well as biological samples including blood, urine, hair, and fingernail clippings. The study directors expect to meet their goal of 100,000 child participants in 2016.

Although the study will span more than two decades, statistically significant data are expected as early as 2012. These early data “will help our understanding of factors related to maternal and child health,” says Kimberly Gray, an epidemiologist and administrator at the NIEHS. “We’ll learn more about reducing pregnancy complications and poor birth outcomes.”

Leaders of the National Children’s Study expect independent researchers to use the data for their own studies, the same way many now do with data from the CDC’s National Health and Nutrition Examination Surveys. “It will stimulate research,” Gray says. “The value will be incredible to the research community.”

